# Viral enteritis after allogeneic hematopoietic stem cell transplantation: pathogens, clinical characteristics, and outcomes

**DOI:** 10.3389/fmed.2025.1638865

**Published:** 2025-08-07

**Authors:** Hai-Lu Sun, Xiang-Yu Zhao, Xiao-Dong Mo, Meng Lv, Yu-Qian Sun, Fang-Fang Wei, Lan-Ping Xu, Yu Wang, Xiao-Hui Zhang, Xiao-Jun Huang, Xiao-Su Zhao, Xu-Ying Pei

**Affiliations:** ^1^Beijing Key Laboratory of Hematopoietic Stem Cell Transplantation, National Clinical Research Center for Hematologic Disease, Peking University People’s Hospital, Peking University Institute of Hematology, Beijing, China; ^2^Peking-Tsinghua Center for Life Sciences, Beijing, China; ^3^State Key Laboratory of Natural and Biomimetic Drugs, Beijing, China

**Keywords:** viral enteritis, allogeneic hematopoietic stem cell transplantation, cytomegalovirus, Epstein-Barr virus, human herpesvirus 6

## Abstract

Viral enteritis is a common complication after allogeneic hematopoietic stem cell transplantation (allo-HSCT). However, data regarding the most frequent enteric pathogens, clinical characteristics, and patient outcomes remains limited. To better characterize post-HSCT viral enteritis, we retrospectively analyzed 59 patients who underwent allo-HSCT and were diagnosised with viral enteritis based on intestinal biopsy specimens. The most frequently identified pathogens were cytomegalovirus (CMV), human herpesvirus 6 (HHV-6), and Epstein-Barr virus (EBV), accounting for 37.3%, 37.3%, and 33.9% of cases, respectively. The median time for diagnosis was 56 days post-allo-HSCT. Diarrhea and abdominal pain were the predominant symptoms. Notably, 35 patients experienced diarrhea lasting 14 days or more, with a median duration of 16 days (range: 3–57 days). Forty-five patients were diagnosed with concurrent graft-versus-host disease (GVHD) by endoscopic examination. The overall survival rates for patients with viral enteritis at 1 and 3 years were 68.7% and 58.1%, respectively. Importantly, patients with CMV enteritis had significantly poorer overall survival compared to those with other viral enteritis types (*P* = 0.035). In conclusion, viral enteritis is a significant complication following allo-HSCT, with CMV, HHV-6, and EBV being the most common pathogens. Early identification and management are crucial, especially for CMV enteritis which is associated with poorer outcomes.

## Introduction

Allogeneic hematopoietic stem cell transplantation (allo-HSCT) is potentially curative treatment for patients with hematologic malignancies. However, viral infections remain a significant challenge after allo-HSCT, contributing substantially to morbidity and mortality among recipients (1). Viral enteritis, characterized by an acute inflammatory response in the intestinal tract triggered by various viruses, constitutes a common complication post allo-HSCT (2).

In contrast to the general population, where rotaviruses and noroviruses are predominant causes of viral enteritis (3), cytomegalovirus (CMV) and Epstein-Barr virus (EBV) are common pathogens in post-HSCT patients. While viral enteritis in healthy individuals is typically self-limiting, resolving within 2–5 days, it can persist for weeks or months in immunocompromised HSCT recipients, often associated with poor transplant outcomes (4–7). Previous study reported by Byung-Sik Cho has identified CMV enteritis as a significant risk factor for non-relapse mortality in patients suffering from intestinal graft-versus-host disease (GVHD) (8). Other reports also highlighted the considerable morbidity caused by norovirus gastroenteritis in immunocompromised HSCT recipients (9–11).

Despite its clinical significance, research specifically addressing post-HSCT viral enteritis remains limited. Early signs and symptoms of post-HSCT viral enteritis are often non-specific, making diagnosis challenging as they can resemble those of bacterial or fungal infections and GVHD (12). The rapid progression of the condition further complicates diagnosis and treatment, necessitating prompt and accurate identification and intervention. This underscores the need for further investigation into the pathogens, clinical presentations, associated risk factors, and prognostic outcomes of viral enteritis in allo-HSCT recipients.

In this retrospective study, we analyzed data from 59 patients diagnosed with viral enteritis by intestinal biopsy specimens after allo-HSCT at Peking University People’s Hospital. Our aim was to describe the frequency of viral pathogen in post-HSCT viral enteritis, and to comprehensively investigate the clinical manifestations, prognostic factors, and mortality rates associated with viral enteritis.

## Materials and methods

### Patients and study design

From January 2016 to December 2018, patients who received allo-HSCT at our center were candidates and were enrolled if they met the following criteria: (i) patient’s age ≥ 14 years, (ii) underwent a colonoscopy because of gastrointestinal symptoms, and had positive viral DNA by polymerase chain reaction (PCR) in intestinal tissue. The Ethics Committee of Peking University People’s Hospital approved this study and informed consent was obtained from each patient. The protocol followed the Declaration of Helsinki ethics guidelines.

### Transplant protocol

Transplant protocols, including the conditioning, mobilization, stem cell harvesting, and acute GVHD prophylaxis protocols were consistent with our previous studies (13–15). Briefly, the main myeloablative preconditioning regimens were cytosine arabinoside (Ara-C), buthionine (3.2 mg/kg/day on days -8, -7, and -6), cyclophosphamide (1.8 g/m2/day on days -5 and -4), and simustine (250 mg/m2 on day -3). The Ara-C dose was 4 g/m2/day in the human leukocyte antigen (HLA)-allogeneic donor (HID) group (days -10 and -9), 2 g/m2/day in the HLA-unrelated donor (URD) group (days -10 and -9), 2 g/m2/day in the HLA-matched sibling donor (MSD) group and 2 g/m2/day (day -9). In addition, the HID and URD groups received rabbit anti-thymocyte globulin (thymoglobulin, 2.5 mg/kg/day on days -5, -4, -3, and -2; Sanofi, France) for the prevention of GVHD. Prophylactic drugs used for mevalonate prophylaxis were mainly cyclosporine A, mycophenolate mofetil, and short-term methotrexate. For steroid-refractory VEGF HD, immunosuppressive therapies such as tacrolimus (FK506), mycophenolate mofetil and baliximab are used.

### Viral monitoring and prophylaxis

Plasma CMV and EBV DNA were monitored by real-time quantitative PCR twice per week in the initial 100 days following allo-HSCT, and followed by once weekly after 100 days, with continued monitoring as deemed clinically necessary (16, 17). Viral DNA loads in plasma over 1000 copies/ml for CMV and over 500 copies/ml for EBV were diagnosed as DNA viremia (17–19). Viruses other than CMV and EBV were not routinely monitored, and were only evaluated for patients with suspicious clinical manifestations.

For viral enteritis monitoring, qPCR was performed using the following commercial kits: (i) For CMV, EBV, ADV, BKV, and JCV, detection was conducted using the Viral DNA Real-Time PCR Kit (Liferiver, Shanghai ZJ Bio-Tech Co., Ltd., Shanghai, China). (ii) For norovirus, rotavirus, and other enteric viruses, detection was conducted using the Viral DNA Real-Time PCR Kit (Tianlong Science and Technology, Suzhou, China). (iii) For HHV-6, HSV-1, B19V, and other respiratory viruses, detection was performed using the Viral DNA Real-Time PCR Kit (Sansure Biotech Inc., Hunan, China). All qPCR amplifications were carried out on the ABI 7500 Real-Time PCR System (Applied Biosystems, USA).

### Diagnosis and definitions

Viral enteritis is defined by the presence of gastrointestinal symptoms and signs along with evidence of viral pathogens in the intestinal mucosa. Diagnostic evidence was obtained via colonoscopic biopsy and confirmed by real-time quantitative PCR testing, The cycle threshold (Ct) values for defining viral positivity were based on the manufacturers’ instructions. To minimize the impact of potential blood contamination on tissue PCR results, all biopsy specimens were collected through colonoscopy under strict hemostatic control and thoroughly washed with saline prior to PCR analysis. The diagnosis of CMV enteritis required both positive immunohistochemical staining for CMV antigens and PCR detection of CMV DNA in mucosal homogenates. EBV enteritis was diagnosed based on positive EBER (EBV-encoded RNA) *in situ* hybridization and PCR detection of EBV DNA in mucosal tissue. For other viruses, comprehensive histopathological confirmation was not available; thus, diagnosis relied on PCR positivity in biopsy samples combined with clinical symptoms and endoscopic findings. The diagnosis of viral enteritis was established at the time the diagnostic specimens, specifically the colonscopy biopsy, are obtained. The definition and grading of acute GVHD is based on the consensus of the Mount Sinai International Consortium for Acute GVHD (20).

### Statistical analysis

Chi-squared and Fisher’s exact test were used to compare categorical variables between cases and controls. The *t*-test was used for the univariate analysis of the continuous variables. Cox proportional hazards regression analysis was used to identify the factors significantly associated with viral enteritis and survival in univariate analysis and multivariate analysis. Kaplan-Meier analysis was performed on mortality data, and curves were compared using the log-rank test. *P*-values were estimated using two-tailed tests, and an α-level of less than 0.05 was regarded as significant. All statistical analyses were performed with SPSS 26.0 (SPSS Inc., Chicago, IL, USA).

## Results

### Patients’ characteristics

Baseline demographic and clinical characteristics of the patients are shown in Table 1. A total of 59 patients were enrolled in our study, of whom 46 underwent haploidentical donor hematopoietic stem cell transplantation (HID-HSCT), 10 received unrelated donor hematopoietic stem cell transplantation (URD-HSCT), and 3 underwent matched sibling donor hematopoietic stem cell transplantation (MSD-HSCT). The median age at transplant was 30 years. A significant proportion (88.1%, *n* = 52) developed acute graft-versus-host disease (GVHD), with 48 patients experiencing grade II-IV severity.

**TABLE 1:** Patients’ characteristics.

	Viral enteritis	
Characteristics	Total (*n* = 59)	CMV(*n* = 22)
**Age (*y*, range)**	30 (14–59)	41.5 (23–55)
**Male (%)**	38 64.4%)	14 (63.6%)
**Underlying disease**		
ALL	22 (37.3%)	11 (50.0%)
AML	18 (30.5%)	5 (22.7%)
CML	2 (3.4%)	0
MDS	11 (18.6%)	3 (13.6%)
AA	3 (5.1%)	3 (13.6%)
Others	3 (5.1%)	0
**Donor-recipient relationship**		
HID-SCT	46 (78.0%)	14 (63.6%)
MRD-SCT	10 (16.9%)	6 (27.3%)
URD-SCT	3 (5.1%)	2 (9.1%)
**Donor-recipient sex**		
Matched	30 (53.6%)	8 (400%)
Mismatched	26 (46.4%)	12 (60.0%)
**Donor-recipient blood type**		
Match	31 (56.4%)	14 (73.7%)
Mismatch	24 (43.6%)	5 (26.3%)
**Engraftment time**		
WBC	14	14
PLT	18	12
**CMV viremia**	50 (84.7%)	18 (81.8%)
**EBV viremia**	27 (45.8%)	7 (31.8%)
**aGVHD**		
Grade II	13 (22.0%)	40 (18.2%)
Grade III	23 (39.0%)	9 (40.9%)
Grade IV	12 (20.3%)	6 (27.3%)

ALL, acute lymphoblastic leukemia; AML, acute myeloid leukemia; CML, chronic myeloid leukemia; MDS, myelodysplastic syndrome; AA, aplastic anemia; HID, haploidentical related donor; MSD, matched sibling donor; URD, matched unrelated donor; WBC, white blood cell; PLT, platelet; aGVHD, acute graft-versus-host disease; CMV, cytomegalovirus; EBV, Epstein-Barr virus.

### Clinical manifestations

The median time from HSCT to diagnosis of viral enteritis was 56 days (range: 28–589 days). In line with the published studies (21, 22), diarrhea (100%, *n* = 59) was common among patients, with a median duration of 16 days (range: 3–57 days) between symptom onset and colonoscopy. Notably, 35 patients experienced diarrhea for more than 2 weeks before diagnosis. The median daily diarrhea volume was 1660 ml. Abdominal pain (71.2%, *n* = 42) and rectal bleeding (49.2%, *n* = 29) were frequent, followed by nausea (13.6%, *n* = 8) and vomiting (13.6%, *n* = 8). Over 60% of patients experienced fever within 2 weeks of colonoscopy. Interestingly, only 10 patients had detectable viruses in peripheral blood compared to 14 patients with viral detection in stool samples.

### Pathological analysis by intestinal biopsy

**FIGURE 1:**
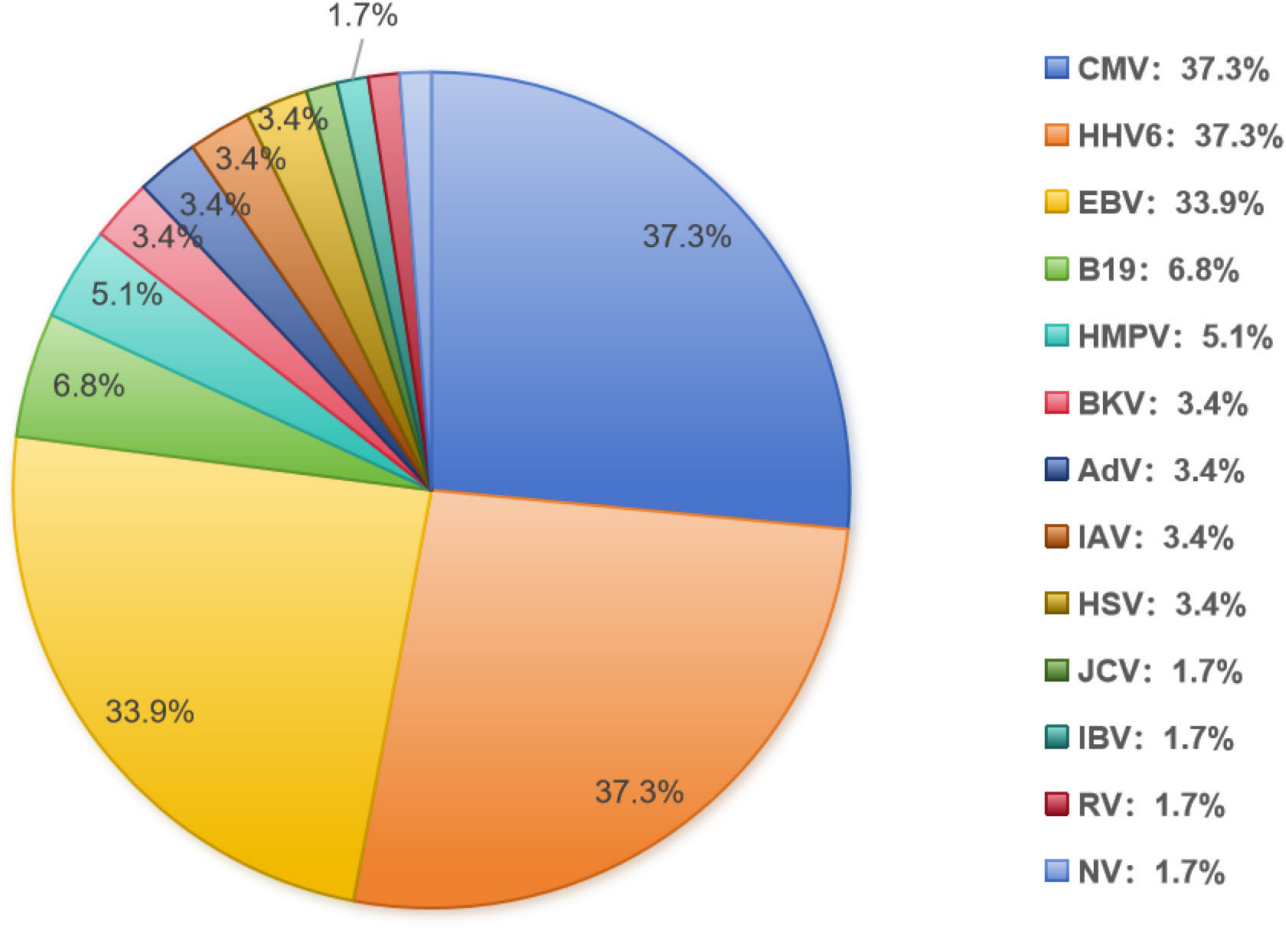
Spectrum and frequency of ausative viruses.

A total of 6 gastroscopies and 65 colonoscopies were performed among the 59 patients. As shown in Figure 1, the most common causes of viral enteritis were CMV and HHV-6, identified in 37.3% of cases. Other viruses were detected with lower frequencies: EBV at 33.9%, followed by parvovirus B19(6.8%), parainfluenza virus (5.1%), BK polyomavirus (3.4%), adenovirus(AdV) (3.4%), influenza A virus H3 (3.4%), herpes simplex virus (HSV) (3.4%), JC polyomavirus, norovirus, influenza B virus, and rotavirus (each identified in just one patient).

CMV was most commonly seen in combination with other viruses, while HHV-6 was most frequently detected alone. Notably, 72.9% of patients had one virus detected based on any positive PCR result at any time post-transplant, 18.6% with 2 viruses, 6.8% with 3 viruses and 1.8% with 6 viruses.

### Impact of timing on viral enteritis characteristics

Patients diagnosed with viral enteritis within 100 days post-transplant experienced significantly serverer higher diarrhea (1947 ml) compared to those diagnosed later (1197 ml; *p* = 0.008). Additionally, a higher incidence of aGVHD was observed in patients with early-onset viral enteritis (within 100 days; 94.9%) compared to those diagnosed later (75.0%; *p* = 0.025). However, the severity of aGVHD (grade III-IV) was similar between the two groups.

**FIGURE 2:**
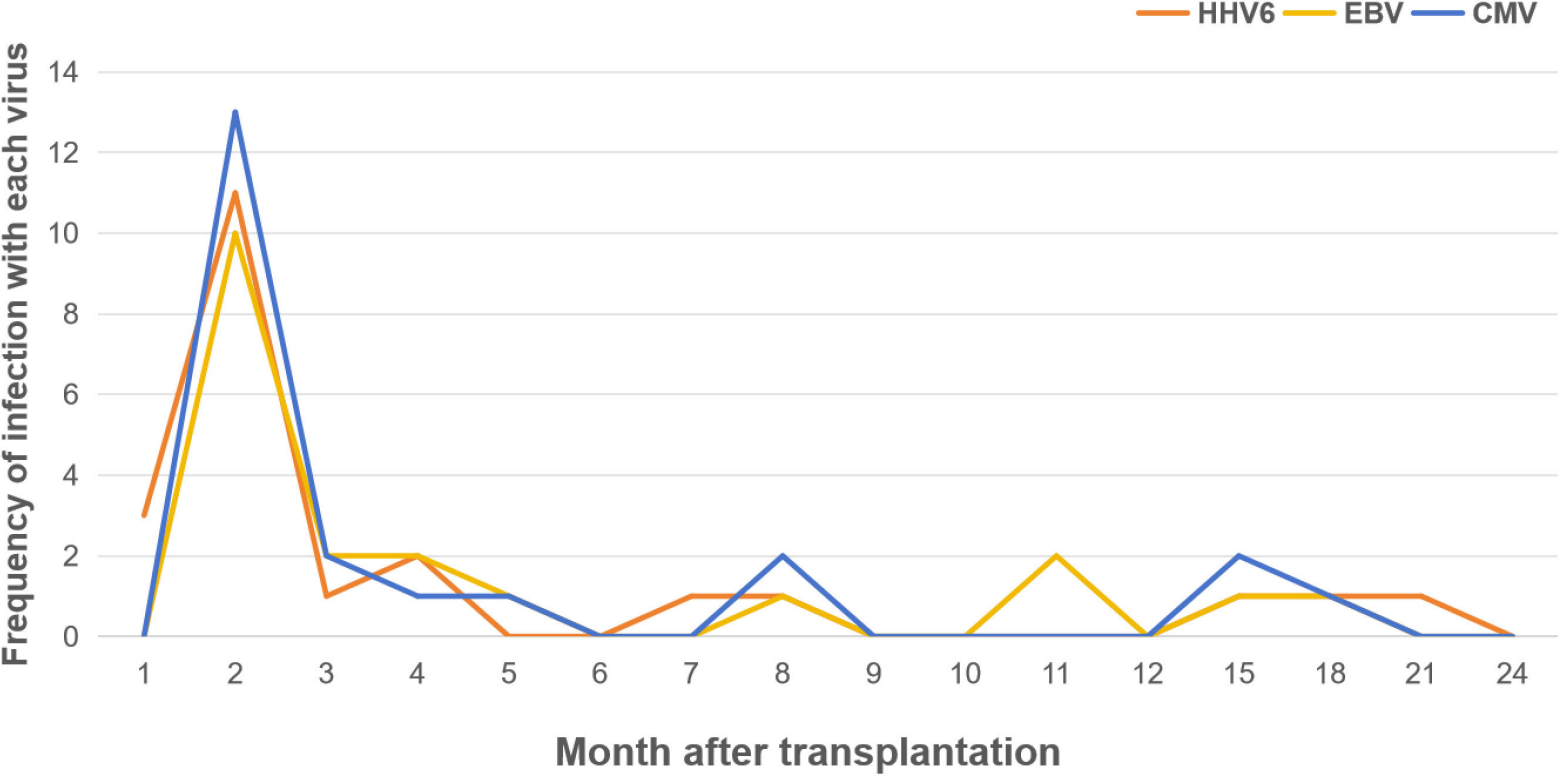
Proportion of patients with detection of HHV6, EBV and CMV enteritis by month post-HSCT.

The type of virus also appeared to be influenced by the timing of diagnosis: The earliest virus to emerge was HHV-6, followed by CMV and BKV. HHV6,CMV,EBV were the most frequently found viral pathogen both within 100 days and beyond after allo-HSCT, as the median diagnosis time for HHV-6 was 45 d after HSCT (range: 28–589 days), the median diagnosis time for CMV was 58 d after HSCT (range: 31–506 days), and 62.5 d for EBV(range: 31–506 days) (Figure 2). The majority of viruses appearing 2 months post-transplantation, and then appearing sporadically until 2 years. However, HSV, rotavirus, and influenza B virus were identified after 100 days of allo-HSCT. In contrast, Norovirus, parainfluenza virus, and polyomavirus were only found within 100 days of allo-HSCT.

### CMV enteritis

Cytomegalovirus enteritis was observed in 37.3% of the patients, with a median diagnosis time of 58 days post-allo-HSCT. Patients with CMV enteritis had a median age of 40.5 years, significantly older than the remaining patients (28 years, *p* = 0.003). For patients with CMV enteritis, the median interval from the onset of diarrhea symptoms to endoscopic diagnosis was 19.5 days, which was longer than the median of 14 days in other patients (*p* = 0.029). Although not significant, the median daily diarrhea volume was larger (2000 ml vs 1200 ml, *p* = 0.359) and rectal bleeding was more common in patients with CMV enteritis (59.1% vs 43.2%, *p* = 0.239).

We routinely monitored serum levels of CMV and EBV in patients post-allo-HSCT and detected fecal CMV copy numbers in patients with gastrointestinal symptoms. To evaluate the correlation between viruses in feces and blood and viral enteritis, we analyzed the data. Only 6 out of 19 patients with CMV enteritis had detectable CMV in their stool samples, while 10 had detectable CMV in their plasma (median copy number: 5.0 × 10^∧^3/mL). Notably, not all patients with detectable CMV in plasma (10 out of 26) were diagnosed with CMV enteritis based on pathology. Similarly, only 6 out of 9 patients with fecal CMV had confirmed CMV enteritis. These findings suggest a weak correlation between CMV detection in stool or plasma and the presence of CMV enteritis as diagnosed by colonoscopy biopsy.

### Transplant outcomes

**FIGURE 3:**
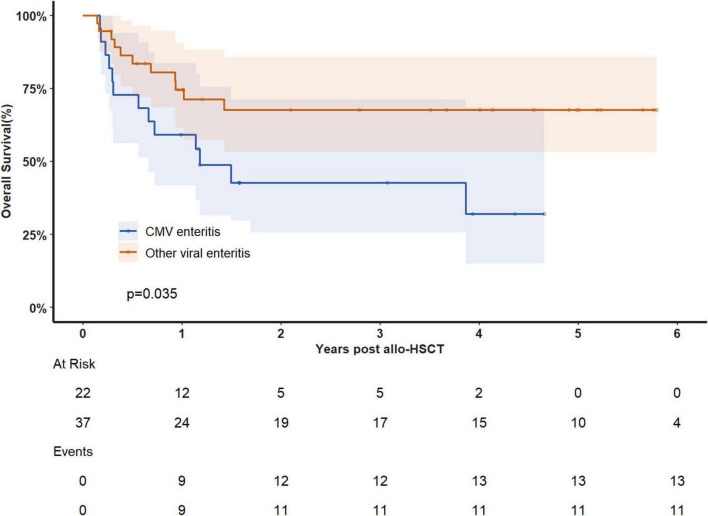
Overall survival analysis in patients with CMV enteritis and other viral enteritis.

The median follow-up time was 4 years, during which 24 patients died. Of these deaths, 21 were attributed to gastrointestinal complications, and 21 resulted from disease recurrence. The overall survival (OS) rate at 180 days after viral enteritis diagnosis was 81.2%(95%CI 71.8–91.9%), decreasing to 68.7% (95%CI 57.6–81.8%) at 1 year and 58.1% (46.2–73.0%) at 3 years. When examining specific viruses, the 1-year OS rates were 57.6% (95%CI 39.9–83.1%) for HHV-6 enteritis, 80.0% (64.3–99.6%) for EBV enteritis and 59.1% (95%CI 41.7–83.7%) for CMV enteritis. Patients with CMV enteritis exhibited significantly lower prognosis compared to those with other viral causes (median OS: 432d vs not reached, *p* = 0.035, Figure 3). We performed Cox regression analyses to evaluate potential risk factors for overall survival (OS), including age, diagnosis, grades III-IV acute GVHD, CMV enteritis, EBV enteritis, and HHV6 enteritis. Univariable analysis identified two significant risk factors, age (HR = 1.045, 95% CI 1.009–1.081, *p* = 0.013), and CMV enteritis (HR = 2.317, 95% CI 1.035–5.186, *p* = 0.041). However, in the multivariable analysis, none of these factors retained statistical significance (Table 2).

**TABLE 2:** Univariate analysis and multivariate analysis of overall survival.

	Univariate analysis			Multivariate analysis		
Characteristics	HR	95%CI	*p*-value	HR	95%CI	*p*-value
Age	1.045	1.009–1.081	0.013	1.03	0.987–1.075	0.171
Diagnosis = AA	0.847	0.114–6.279	0.871	1.723	0.187–15.872	0.631
aGVHD III-IV	1.991	0.824–4.810	0.126	2.244	0.851–5.920	0.102
CMV enteritis	2.317	1.035–5.186	0.041	1.85	0.680–5.032	0.228
EBV enteritis	0.738	0.305–1.785	0.501	0.945	0.350–2.551	0.911
HHV6 enteritis	1.560	0.697–3.492	0.280	2.337	0.861–6.339	0.085

AA, aplastic anemia; aGVHD, acute graft-versus-host-disease; CMV, cytomegalovirus; EBV, Epstein-Barr virus; HHV6, human herpesvirus 6.

## Discussion

In this retrospective study, we analyzed data from 59 allo-HSCT patients diagnosed with viral enteritis through intestinal biopsy specimens. Our findings provide insights into the cumulative incidence, causative viral spectrum, and clinical characteristics of viral enteritis in this population.

Previous research on gastrointestinal infections after HSCT primarily focused on CMV enteritis, emphasizing its impact on clinical outcomes, particularly in patients with GVHD (8, 23–25). However, other viral causes of enteritis have been scarcely described. Our study addresses this gap by identifying 13 different viruses associated with enteritis after HSCT, with a median diagnosis time of 56 days post-transplant. Common symptoms included diarrhea, abdominal pain, bloody stools, and fever. CMV, HHV-6, and EBV were the most frequent viral culprits. Notably, CMV was most commonly seen in combination with other viruses, while HHV-6 was most frequently detected alone. Multiple viral infections were also common, with 27.1% of patients having at least 2 viruses identified in biopsies. Additionally, 8.6% had 3 or more viruses detected, and 1.8% had 4 viruses. Most cases of viral enteritis occurred within 2–3 months after transplantation, with sporadic cases observed up to 2 years post-transplant.

This study identified HHV-6 as a significant cause of viral enteritis following HSCT. While HHV-6 is a known cause of encephalitis after HSCT, its role in enteritis has rarely been reported. Only few small-scale retrospective studies reported the incidence of HHV6 intestinal infection after transplantation (26–28). Previous studies by Perruccio et al. (29) reported that HHV6-related enteritis was observed only in 3.3% of the 123 pediatric patients post HSCT. Ji et al. (30) reported a 2.2% incidence of HHV6-related enteritis in admitted patients within 90d after HSCT. In our study, HHV6 enteritis accounted for 37.3% of viral enteritis, and more than 60% were diagnosed within 90d after HSCT. This suggests HHV6 is an important pathogen for viral enteritis in the early stage after HSCT.

Similar to previous studies (5–8, 21–25, 31, 32), CMV emerged as a significant enteric pathogen on our cohort. Consistent with earlier research by Cho et al. (8), CMV enteritis was associated with increased non-relapse mortality and negatively impacted GVHD-specific survival. Akahoshi et al. (28) also reported a link between CMV gastroenteritis and elevated non-relapse mortality. In our study, patients with CMV enteritis exhibited significantly worse prognoses compared to those with other viral enteritis types. Older age and longer delays between diarrhea symptoms and colonoscopy may be associated with poorer outcomes in CMV enteritis.

While we tested for CMV in peripheral blood and feces, we found no significant correlation between CMV presence in these samples and the diagnosis of CMV enteritis. This aligns with the findings of Sun et al. (32), who reported that fecal CMV DNA is not a reliable predictor for CMV enteritis. In contrast, Gu et al. (23) suggested that digital PCR detection of CMV DNA in cell-free DNA from stool supernatant is a powerful method for identifying CMV gastroenteritis. These discrepancies might be due to differences in the time between stool collection and biopsy, as well as variations in detection methods.

This study has several limitations. First, as a retrospective study, only patients who underwent colonoscopy for suspected viral enteritis were included, potentially missing patients with atypical symptoms. Second, for viruses other than CMV and EBV, additional histopathologic methods such as *in situ* hybridization or immunofluorescence were not routinely performed. Though we diagnose viral enteritis depended on PCR positivity in biopsies alongside clinical-endoscopic correlation, future prospective studies should incorporate multimodal pathogenic evidence detection to improve diagnostic specificity.

In conclusion, this study demonstrates that viral enteritis is a serious complication after HSCT. Our findings provide the first comprehensive analysis of the cumulative incidence, causative viral spectrum, and clinical characteristics of viral enteritis in this population. Additionally, our results suggest a poorer prognosis for patients with CMV enteritis compared to other viral causes. This study significantly contributes to our understanding of diagnosing and managing viral enteritis after HSCT. Future research with larger, prospective cohorts is needed to further explore the risk factors, optimal diagnostic approaches, and treatment strategies for this complex complication.

## Data Availability

The raw data supporting the conclusion of this article will be made available by the authors, without undue reservation.
